# Accuracy–Efficiency Trade-Off: Optimizing YOLOv8 for Structural Crack Detection

**DOI:** 10.3390/s25133873

**Published:** 2025-06-21

**Authors:** Jiahui Zhang, Zoia Vladimirovna Beliaeva, Yue Huang

**Affiliations:** Institute of Civil Engineering and Architecture, Ural Federal University, St. Mira19, 620002 Yekaterinburg, Russia; imhuangyue@163.com

**Keywords:** YOLOv8, crack detection, attention mechanism, SimAM, C3Ghost, feature pyramid, accuracy–efficiency trade-off

## Abstract

To address the accuracy–efficiency trade-off faced by deep learning models in structural crack detection, this paper proposes an optimized version of the YOLOv8 model. YOLO (You Only Look Once) is a real-time object detection algorithm known for its high speed and decent accuracy. To improve crack feature representation, the backbone is enhanced with the SimAM attention mechanism. A lightweight C3Ghost module reduces parameter count and computation, while a bidirectional multi-scale feature fusion structure replaces the standard neck to enhance efficiency. Experimental results show that the proposed model achieves a mean Average Precision (mAP) of 88.7% at 0.5 IoU and 69.4% for mAP@0.5:0.95, with 12.3% fewer Giga Floating Point Operations (GFlops), and faster inference. These improvements significantly enhance the detection of fine cracks while maintaining real-time performance, making it suitable for engineering scenarios.

## 1. Introduction

With the acceleration of urbanization and the extension of building service life, concrete structures are increasingly exposed to sustained loads, environmental factors, temperature changes, and other conditions, leading to the gradual formation of surface cracks. These cracks not only compromise the appearance and functionality of the structures but also pose serious risks to their safety and durability. Statistics indicate that approximately 60% of in-service buildings exhibit varying degrees of cracking [1], making the efficient and accurate detection of structural cracks a critical research focus in civil engineering.

Traditional crack detection methods mainly include manual inspection [2], sensor-based monitoring [3], image processing techniques (such as edge detection and threshold segmentation) [4], and conventional machine learning algorithms (e.g., SVM, KNN) [5]. However, manual inspection suffers from low efficiency and high subjectivity; sensor-based detection is costly and requires expert operation; and image processing approaches often lack robustness in complex backgrounds.

In recent years, the emergence of deep learning has provided new avenues for automated crack detection. Convolutional Neural Networks (CNNs), with their powerful feature extraction capabilities, enable rapid localization and identification of cracks [6]. For instance, Laxman et al. proposed a deep learning framework that not only detects cracks but also predicts their depth, thereby enhancing the practicality of structural health monitoring [7].

As a representative single-stage object detection algorithm, the YOLO series has been widely adopted in crack detection tasks due to its end-to-end architecture and real-time performance. In particular, the YOLOv8 model released by Ultralytics in 2023 has achieved a favorable balance between detection accuracy and computational speed. However, the direct application of YOLOv8 to crack detection still faces several challenges:

1. Crack patterns are irregular, and complex backgrounds hinder effective feature extraction;
2. Fine crack features are easily lost in deeper network layers;
3. High-precision models often have significant computational demands, making them unsuitable for embedded deployment.

To address these challenges, recent studies have explored various optimizations of the YOLOv8 architecture. For example, Dong et al. integrated attention mechanisms to enhance detection precision, but this significantly increased model size (up to 12 M parameters), limiting its deployment on edge devices [8]. Ren et al. improved fine crack detection using deep networks, but at the cost of much slower inference speeds [9]. Li et al. proposed a lightweight variant of YOLOv8n to improve efficiency; however, it still struggled with detecting small cracks [10].

In response to these limitations, this study proposes an optimized, lightweight YOLOv8n model aimed at improving both detection accuracy and computational efficiency. The main innovations are as follows:

1. Integration of the parameter-free SimAM attention mechanism to enhance key feature responses;
2. Adoption of the C3Ghost module to replace standard convolution layers, reducing model complexity and parameter count;
3. Design of a Concat_BiFPN multi-scale feature fusion structure to improve the detection of fine cracks;
4. Development of a self-constructed dataset with 1959 images, covering scenes such as concrete, roads, and tunnels;
5. Validation of the proposed method’s generalization and superiority on public benchmark datasets.

Through these optimizations, the proposed method significantly enhances micro-crack detection while maintaining real-time performance, offering an efficient and reliable technical solution for structural health monitoring in civil engineering.

## 2. Related Work

### 2.1. Development of Crack Detection Methods

The development of structural crack detection methods has evolved from traditional visual inspection to machine learning-based automatic detection, and finally to the application of deep learning techniques. Early studies mainly utilized image processing techniques such as edge detection and threshold segmentation. Although these methods were simple to implement, their performance significantly deteriorated under complex backgrounds. For example, Gupta et al. systematically reviewed image-based crack detection methods and pointed out that traditional algorithms (such as Canny edge detection and morphological processing) show notably decreased performance when faced with uneven lighting and background interference [4].

With the advancement of machine learning, classifiers based on handcrafted features, such as SVM and KNN, were introduced into crack detection. As shown in early research by Zhang et al., adaptive threshold segmentation combined with SVM could achieve crack recognition in simple backgrounds, but it required strong feature representation and showed limited generalization in complex environments [5]. Meanwhile, Yang et al. proposed an improved Mask R-CNN for micro-crack detection, which significantly enhanced accuracy and small object recognition compared to the original model [11].

In the field of object detection, the YOLO (You Only Look Once) series has been widely used in crack detection tasks due to its end-to-end structure and real-time performance. Since 2019, Ultralytics has driven the development of YOLOv8, improving detection speed and accuracy. However, directly applying YOLOv8 to crack detection still faces challenges, especially in scenarios with small targets and complex background interference, where accuracy drops significantly. To address this, Alshawabkeh et al. combined Mask R-CNN with Vision Transformer and proposed a hybrid method that achieved superior recall performance on the DeepCrack dataset [12].

Additionally, Yang et al. introduced the DeepCrack framework, which was the first to apply FCN for pixel-level crack segmentation [13]. Chen et al. improved Mask R-CNN to support multi-class crack recognition, significantly enhancing semantic segmentation capability and detection robustness [14]. Wang et al. emphasized the pixel-level accuracy of FCN architecture for multi-scale detection in combination with YOLO’s detection framework for fast localization of road cracks [15]. We summarize the development timeline of crack detection technologies (Table 1) and performance comparison of typical algorithms (Table 2).

### 2.2. Improvements of the YOLO Series Models

At the beginning of 2023, Ultralytics released the YOLOv8 model, which features a five-size architecture (YOLOv8n/s/m/l/x) designed to accommodate detection tasks of varying complexity. Structurally, YOLOv8 replaces the coupled head used in YOLOv5 with a decoupled head to better separate classification and regression pathways. It also adopts Complete IoU (CIoU) as the loss function for bounding box regression and introduces the C2f module to replace the traditional C3 structure. This contributes to a notable enhancement in the model’s ability to capture and express relevant features [25].

With these structural optimizations, YOLOv8 demonstrates a clear advantage in balancing speed and accuracy. Therefore, this study selects YOLOv8n as the baseline model to conduct lightweight research aimed at edge computing scenarios. Wu et al. introduced the CBAM (Convolutional Block Attention Module) into YOLOv8, significantly improving crack detection accuracy; however, CBAM involves learnable parameters, which add considerable computational overhead [26].

Building on this, Zhang et al. incorporated a Transformer encoder into YOLOv7 to enhance small object detection performance. Although this approach improved detection capabilities, experiments showed a significant drop in real-time performance, making it unsuitable for resource-constrained platforms [27].

To strike a balance between real-time performance and accuracy, the SimAM (Simple Attention Module) has garnered attention. SimAM does not require learnable parameters and enhances key channel responses based on neuro-cognitive heuristics. Studies have shown that SimAM can improve detection performance without increasing model complexity, making it an ideal attention mechanism for lightweight applications [28].

### 2.3. Model Selection

The choice of YOLOv8 as the baseline model is based on the following considerations:

1. Excellent Balance Between Detection Performance and Efficiency

YOLOv8 shows significant detection accuracy improvement over YOLOv5 and YOLOv7 on the COCO dataset (Common Objects in Context) [29], which is a standard common target detection dataset released by Microsoft and contains a wide range of everyday object classes (80 classes). YOLOv8 optimizes the network structure and computation process and improves the inference speed by about 10–20% on the same hardware platform, which is especially suitable for embedded or mobile applications with high real-time requirements [30].

2. Innovative Architectural Design

YOLOv8 abandons traditional anchor-based detection methods and adopts an anchor-free architecture, reducing the complexity of hyperparameter tuning [31]. Its backbone utilizes an improved CSPDarkNet structure with cross-stage connections to enhance feature extraction efficiency. The neck incorporates an optimized PANet, improving the detection performance for small objects [32]. Furthermore, YOLOv8 introduces mechanisms such as the Task-Aligned Assigner, which dynamically allocates labels during training to improve both training efficiency and detection accuracy [33].

3. Flexible Model Scaling and Lightweight Support

Ultralytics offers five model scales—YOLOv8n, YOLOv8s, YOLOv8m, YOLOv8l, and YOLOv8x—allowing users to flexibly choose between detection accuracy and resource consumption according to specific application needs [34].

YOLOv8 includes five official model variants—n, s, m, l, and x. Based on a comprehensive consideration of various factors, we selected YOLOv8n as the baseline model due to its compact size and fast inference speed. Its relatively shallow depth and narrow width make it a more convenient and efficient choice for deployment. The performance comparison of different sizes of YOLOv8 models on the COCO dataset is shown in Table 3.

Furthermore, YOLOv8 demonstrates strong applicability to pavement crack detection tasks. Pavement cracks are typically narrow and irregular and often appear under complex backgrounds with low contrast, which makes them difficult to detect accurately. YOLOv8’s anchor-free design, enhanced feature extraction (via the C2f module), and its ability to capture multi-scale features contribute to robust detection performance, especially for small and elongated defects. In addition, its lightweight architecture enables efficient inference, making it suitable for real-time crack detection scenarios, including deployment on mobile or edge devices in road inspection systems.

### 2.4. Image Input

Due to the varying aspect ratios among different images in the dataset, the YOLOv8 algorithm implements a series of preprocessing steps aimed at optimizing the scaling of input images. This mechanism enables images to adapt to the standard input size of 640 × 640 pixels while minimizing unnecessary padding with black borders and improving anchor box matching efficiency [36]. YOLOv8 integrates mosaic data augmentation, which scales and stitches together four images to greatly enhance sample diversity and detection robustness [37].

During training, YOLOv8 first generates predicted bounding boxes based on initial anchor frames, compares them with ground truth boxes, and continuously optimizes parameters by minimizing the loss through backpropagation [38].

The YOLOv8 algorithm consists of four main components: input, backbone, and neck. The YOLOv8 framework is composed of four key modules: the input layer, backbone, neck, and detection head. The input module is specifically designed to handle high-resolution images, which is essential for capturing the fine-grained details of civil structural cracks. The backbone employs advanced multi-scale feature extraction techniques, enabling the network to effectively manage variations in crack size and shape. The neck component fuses hierarchical features across different scales, thereby enriching the feature representation and enhancing the sensitivity to crack morphology. Finally, the detection head incorporates an adaptive anchor mechanism that dynamically adjusts anchor dimensions to align with the characteristics of civil structural crack datasets. This design significantly improves the accuracy of crack localization and classification, providing critical support for subsequent maintenance and structural assessment tasks.

**Figure 1:**
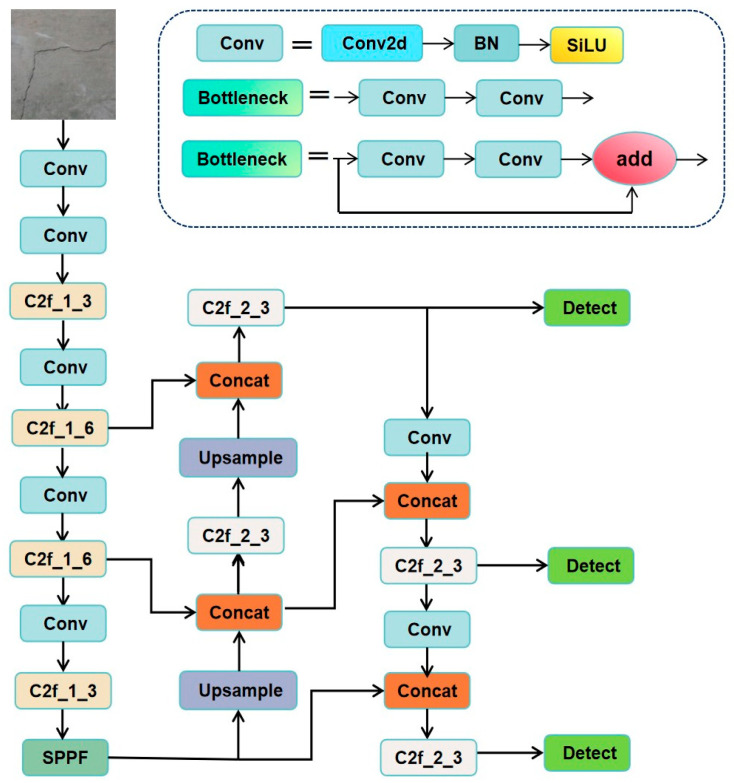
YOLOv8n network architecture diagram (Conv: standard convolutional layer (convolution + BN + SiLU activation); C2f: cross-stage feature fusion module (replacing the original C3 structure) to enhance gradient flow and multi-scale feature learning; SPPF: Spatial Pyramid Pooling Fast Module, preserving contextual information through multi-scale pooling; Concat: feature splicing operations; Upsample: upsampling layer; Detect: detection header (classification + regression branch decoupling)).

A visual representation of the YOLOv8 algorithm’s architecture layout is provided in this paper (Figure 1).

### 2.5. Backbone Network

In the YOLOv8 algorithm, the backbone network plays a crucial role in extracting general features of target objects. This network is composed of three main modules: Conv, C2f, and SPPF. The Conv module includes the Conv2d operation, Batch Normalization (BN), and SiLU activation functions. It employs an autopad (k, p) mechanism to achieve padding, effectively addressing the issue of blurred edges in bridge cracks [39]. The C2f module is a hybrid design derived from the fusion of the C3 module and ELAN (Efficient Layer Aggregation Network) architecture. Through a multi-branch structure, it enhances gradient flow and improves the learning capability for features at different scales and abstraction levels. This module not only boosts nonlinear modeling capacity but also significantly increases sensitivity to small crack detection [40].

**Figure 2:**
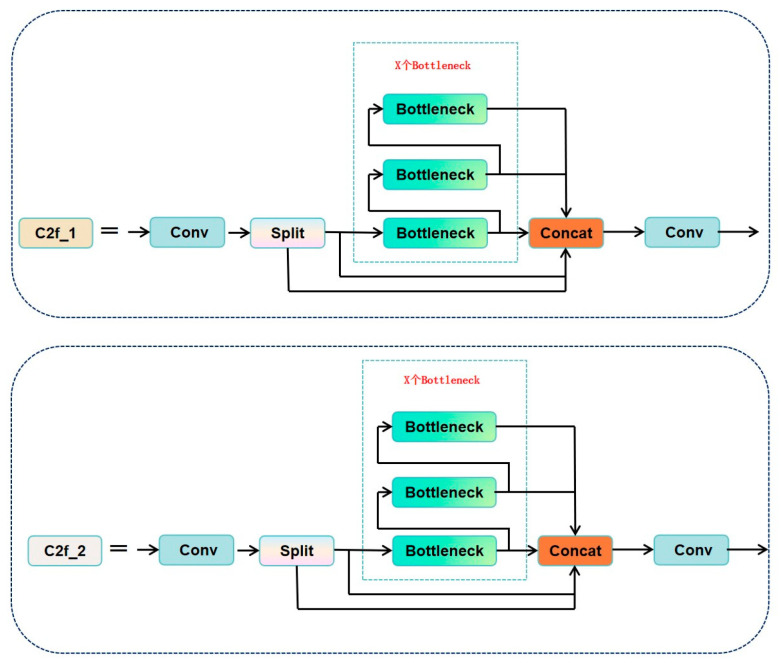
C2f module: contains bottleneck stacking and cross-layer connections.

**Figure 3:**
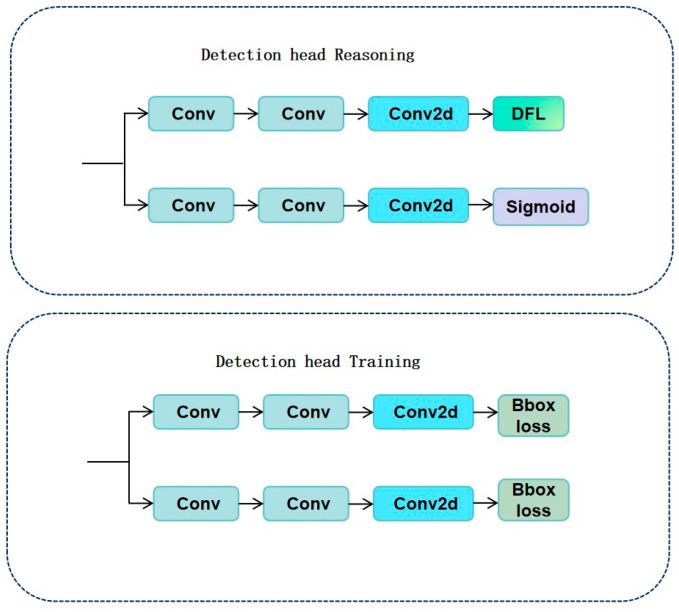
Detection module: decouples classification and regression headers.

**Figure 4:**
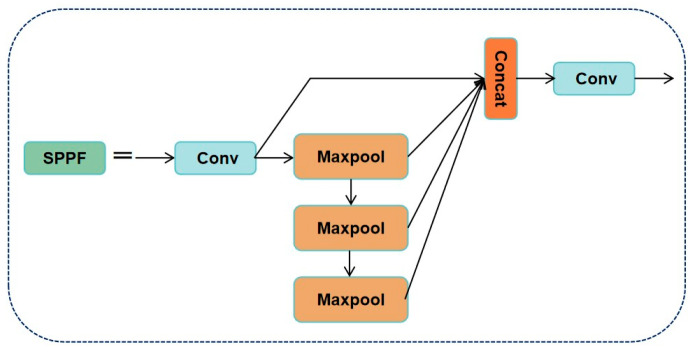
SPPF module: tandem small pooled kernels instead of a single large kernel.

Further studies indicate that this structure is especially suitable for multi-scale modeling of crack-like targets in bridges or earthquake-damaged components [41]. The SPPF module (Spatial Pyramid Pooling Fast) is a redesign of the original SPPNet in YOLOv8. By cascading multiple small pooling kernels to replace the original large-scale kernel, it retains multi-scale contextual awareness while optimizing computational efficiency. This module enhances the model’s robustness in handling cracks of various sizes, such as transverse and longitudinal micro-cracks on bridge decks [42]. The structures of the C2f, detection module, and SPPF modules are illustrated in Figure 2, Figure 3 and Figure 4.

### 2.6. Neck

In the YOLOv8 algorithm, the neck network skillfully combines the concepts of FPN (Feature Pyramid Network) and PAN (Path Aggregation Network) to create an advanced FPN + PAN hybrid structure. This module serves as a link between the backbone and the head, playing a vital role in the fusion and reconstruction of multi-level features [43].

In this structure, FPN transmits semantically enhanced high-level features from top to bottom to improve object discrimination, while PAN uses a bottom-up approach to feed spatially precise low-level features back into the high-level semantic flow, enhancing the hierarchical consistency of overall feature representation [44]. This bidirectional information flow strategy significantly optimizes robustness for detecting small objects (such as cracks) and complex backgrounds.

Moreover, compared to YOLOv5, the YOLOv8 neck network abandons the convolutional structures in the original upsampling stages, simplifying channel operations to reduce inference latency while retaining PAN’s ability to respond to fine textures. This lightweight architectural improvement is widely applied in road defect and surface crack detection fields [45].

### 2.7. Head

The YOLOv8 algorithm adopts a decoupled head structure, which separates classification and regression tasks structurally by designing distinct classification and detection heads. This separation enhances their respective expressive capacities and task robustness across different target dimensions. Compared to YOLOv5’s coupled detection head, this design helps alleviate gradient interference between multi-task learning, improving detection accuracy and training convergence speed [46].

Specifically, YOLOv8 deploys three detection layers at different resolutions in its head, each connected to feature maps from the neck network to detect multi-scale objects. An adaptive anchor design dynamically adjusts the size and shape of anchors to better fit varying object structures [47].

To enhance regression accuracy, YOLOv8 introduces Distribution Focal Loss (DFL). Compared to traditional GIoU or DIoU losses, DFL provides smoother and more informative localization gradients, particularly suited for dense object localization tasks [48].

Additionally, the model employs an anchor-free architecture during detection output, avoiding reliance on preset static anchors. Instead, it directly regresses bounding boxes through feature position regression. This mechanism, combined with the decoupled head and DFL, significantly improves detection performance in complex scenarios such as cracks and mechanical surface defects [49].

## 3. Improved YOLOv8 Model Design

### 3.1. Overall Architecture

**Figure 5:**
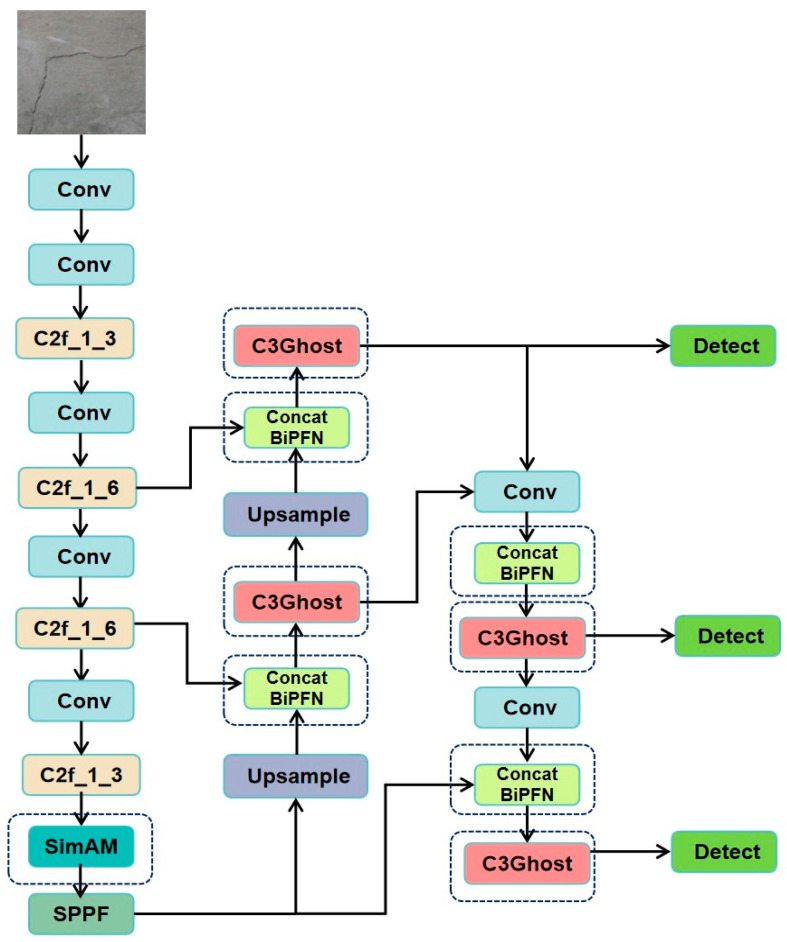
Improved YOLOv8 network architecture diagram.

As shown in Figure 5, the improved model consists of three core modules: (1) A SimAM module is added after the last C2f_1_3 module in the backbone network and before the SPPF module to enhance the channel and spatial weights (parameter-free attention) of crack features, thereby improving the response capability for low-contrast cracks; (2) the C2f module in the neck (Neck) is replaced with the C3Ghost module, the Concat module is replaced with the Concat_BiFPN module, and standard convolutions are replaced with Ghost convolutions (1 × 1 standard convolution + 5 × 5 depth convolution) to reduce computational complexity while maintaining feature representation capability; (3) the detection head (Head) retains its original design. The total number of parameters is 2.51 million, a reduction of 0.49 million compared to the original model, representing a decrease of 16.33%.

### 3.2. SimAM Attention Mechanism (Parameter-Free Attention)

**Figure 6:**
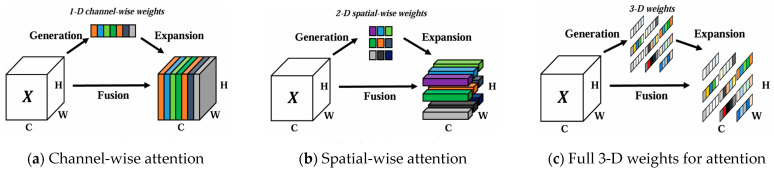
Comparisons of the different attention modules.

SimAM (Simple Attention Module) [28] (Figure 6) is a parameter-free attention mechanism that dynamically enhances key feature responses in deep networks by simulating the “attention” and “focus” processes of the human visual system, without introducing additional learnable parameters. Attention mechanisms have been widely applied in fields such as computer vision, natural language processing, and multimodal learning, with the core goal of improving a model’s adaptive perception of information focus.

In the field of object detection, research shows that integrating SimAM can effectively enhance the model’s representation of target regions while suppressing background and non-target interference, thereby improving detection accuracy and robustness [50]. SimAM evaluates the “importance” of neurons through a neural energy model, highlighting responses in visually salient regions. Without any learnable parameters, it significantly boosts the model’s ability to recognize key areas [51].

In applications such as remote sensing detection, object tracking, and street scene segmentation, the integration of SimAM into backbone networks like YOLO or RepVGG has notably improved recognition accuracy for small targets and edge regions [52]. Additionally, SimAM has been incorporated into mainstream detectors like YOLOv5 and YOLOv8, enabling precise perception of complex targets such as power grid equipment and road cracks [53].

Traditional attention modules like SE and CBAM require extra learnable parameters for feature weights. SimAM achieves more efficient attention modeling through an energy function:

The feature weighting in SimAM is defined as shown in Equation (1):

(1) $$F_{A}(i,j,c)=F(i,j,c)\cdot A(i,j)$$

Among these, *F_A_*(*i*,*j*,*c*) is an element of the input feature map, and *A*(*i*,*j*) is the corresponding attention weight. In Equation (1), each element of the input feature map is multiplied by its corresponding attention weight to obtain the weighted feature map *F_A_*. This process does not require additional parameters and only involves element-level multiplication operations, resulting in lower computational complexity and higher efficiency compared to traditional attention mechanisms. Second, SimAM is designed to be lightweight, with all operations being highly efficient and requiring no additional parameters or complex computational steps. This means that while improving model performance, SimAM maintains extremely high computational efficiency. The attention weights *A*(*i*,*j*) in SimAM are generated through feature energy normalization, which involves two steps:

(1) Channel energy calculation, as shown in Equation (2):

(2) $$S(i,j)=\sum_{c=1}^{c}F{\left(i,j,c\right)}^{2}$$

Among them, *c* is the number of feature channels, and *S*(*i*,*j*) represents the channel energy at spatial position (*i*,*j*) (quantified by the sum of the squares of the eigenvalues of the channel dimension, which quantifies the visual significance of the region).

(2) Energy normalization, as shown in Equation (3):

(3) $$A(i,j)=\overset{\wedge }{S}(i,j)=\frac{S(i,j)}{\sum_{\left(i,j\right)}S(i,j)}$$

After normalization, *Ŝ*(*i*,*j*) (*A*(*i*,*j*)) represents the “contribution probability” of spatial position (*i*,*j*) to object detection, which is directly used as the attention weight in Equation (1).

Through the complete chain of channel energy aggregation (Equation (2)) → probability normalization (Equation (3)) → feature weighting (Equation (1)), SimAM enables the model to adaptively focus on visually salient regions without parameters. This mechanism ensures computational efficiency while enhancing feature representation capabilities, ultimately validating its ability to improve crack detection accuracy in ablation experiments.

Principle: The energy function-based participantless attention mechanism dynamically enhances the feature map by modeling the saliency of neurons. At its core, it automatically learns the weights of each channel by minimizing the energy function without additional trainable parameters.

Advantage: Enhancement of small target features: In crack detection, SimAM can focus on thin, low-contrast crack regions to enhance localized feature response.

Zero number of parameters: no increase in model complexity, suitable for lightweight scenarios.

### 3.3. C3Ghost Module Optimization

**Figure 7:**
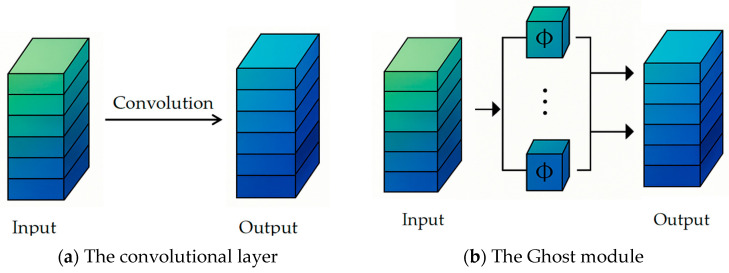
Process of Traditional Convolution vs. Ghost Convolution.

GhostNet, proposed by Huawei Noah’s Ark Lab, is a novel lightweight neural network architecture distinct from MobileNet and ShuffleNet. Its goal is to generate feature maps more efficiently, reducing redundant computations caused by traditional convolutions. In GhostNet, the Ghost module first extracts intrinsic feature maps using 1 × 1 convolutions, then cheaply generates additional complementary feature maps through 5 × 5 linear transformations (Figure 7), and finally concatenates both to form a complete feature set [54].

**Figure 8:**
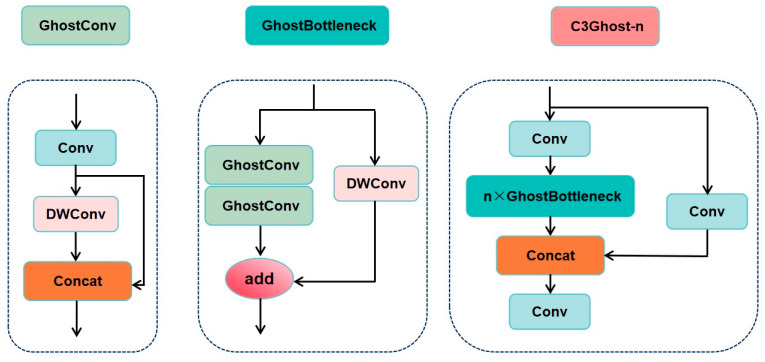
C3Ghost module architecture diagram (GhostConv: lightweight convolutional layer (generating essential features by 1 × 1 convolution+phantom features by deep convolution); GhostBottleneck: residual bottleneck structure (two GhostConv + Add operation); *n* × GhostBottleneck: cascade *n* GhostBottleneck modules; Concat: feature splicing operation (preserves features of original and processed paths); Conv: standard convolutional layer (fuses spliced features)).

This design achieves three key advantages: reduced model complexity, insensitivity to feature map size, and compression of parameter count, resulting in significant performance gains in edge computing and embedded scenarios [55]. Moreover, in lightweight improvements of the YOLO series, the fusion of the Ghost module with the C3 structure—called the C3Ghost module (Figure 8)—has been demonstrated to reduce over 30% of parameters and FLOPs while maintaining nearly unchanged detection performance [56].

Feature compression: Ghost convolution is used to split standard convolution into two steps:

The first step uses a small number of 1 × 1 convolutions to generate “intrinsic features”, significantly reducing the number of parameters.

The second step uses linear transformations to generate “ghost features”, which supplement the detailed information.

Feature Retention: Cross-stage connections (C3 structure) are used to retain multi-scale gradient flows, preventing shallow-layer features from being lost during compression. Experiments show that the improved model mAP@0.5:0.95 improved by 2.1%, verifying its ability to retain small crack features.

Studies also show that integrating GhostConv and C3Ghost modules in various applications, such as wood defect detection and low-light target detection, achieves very high computational efficiency and feature preservation [57,58].

Traditional convolutional operation flow, see Figure 7a: The convolutional layer: Input feature maps are fully connected computed by dense convolutional kernels, each output channel needs independent convolutional kernel parameters, and the complete output feature maps are generated directly, and lightweight Ghost convolutional flow, see Figure 7b. The Ghost module: Input feature maps are first generated through the 1 × 1 convolution to generate the essential feature map, the essential features are transformed by cheap linear transformation to generate the phantom feature map, and the essential features are spliced with the phantom features to form the final output.

Principle: The standard convolution in the C3 module is replaced by C3Ghost convolution, which generates redundant feature maps by a linear transformation, reducing computation. Ghost convolution splits the input channel into two parts: one part is convolved conventionally, and the other part generates “phantom” features by low-cost operations (e.g., deep convolution).

Advantage: 16.33% reduction in parameterization: The introduction of the C3Ghost module reduces the number of model parameters from 3.0 M to 2.51 M.

Maintaining feature expressiveness: Avoiding significant degradation of accuracy through a feature reuse strategy.

### 3.4. Concat_BiFPN Feature Fusion

**Figure 9:**
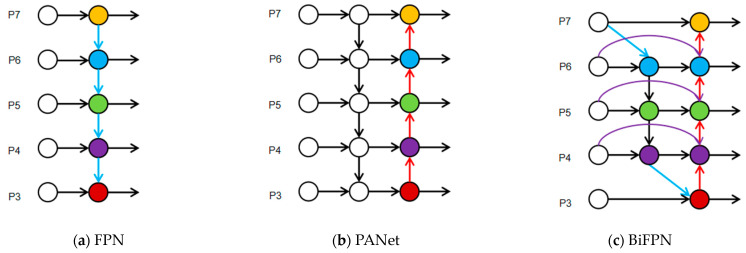
Neck feature network design (red arrows: top-down path (high semantic features to low level); blue arrows: bottom-up path (low-level spatial details to high level); green module: feature fusion node (weighted summation); structural comparisons: (**a**) FPN adopts top-down information flow (blue arrows) to transfer high-level semantic information to low-level features. (**b**) PANet introduces bottom-up paths (red arrows) on the basis of FPN to enhance the expression of low-level features. (**c**) BiFPN simultaneously fuses top-down (red arrows) and bottom-up (blue arrows) paths and adds cross-layer connections (purple arrows) to achieve efficient and weighted multi-scale feature fusion.

The Concat_BiFPN module plays a crucial role in efficient multi-scale feature fusion for object detection tasks (Figure 9). This structure combines the bidirectional cross-scale connection mechanism of BiFPN (Bidirectional Feature Pyramid Network) with the channel concatenation strategy of Concat, significantly enhancing the information exchange between deep and shallow features. Studies have shown that BiFPN effectively improves feature reuse efficiency, avoiding the uneven top-down or bottom-up information flow problems seen in traditional FPNs, and supports iterative multi-level semantic fusion [59]. The bidirectional pathway design (Top-Down + Bottom-Up) employs a soft weighting mechanism to adaptively fuse semantic layers at different scales, further boosting the network’s responsiveness to small objects [60].

Core Mechanism and Scale Optimization:

1. Scale Adaptability of Bidirectional Paths

Bottom-Up Path: Strengthens shallow high-resolution features and specifically optimizes the retention of small-scale crack details, avoiding feature blurring of small targets caused by deep subsampling through cross-layer connections.

Top-Down Path: Transmits high-semantic features from deeper layers to enhance background suppression capabilities for large-scale cracks, reducing interference from complex textures (e.g., concrete surfaces) on large crack detection.

2. Dynamic Weighting and Scale Balancing

Employs a learnable weighting mechanism with Softmax normalization to adaptively allocate weights based on the importance of features at different scales: Small-scale cracks rely on shallow-layer details, so weights are tilted toward lower-level features; large-scale cracks rely on deep-level semantics, so weights are tilted toward higher-level features; medium-scale cracks achieve balanced weights through bidirectional fusion.

Compared to traditional FPN, Concat_BiFPN uses Softmax for feature weighting normalization, which significantly alleviates training blockages caused by gradient instability while ensuring the importance of each feature layer in the flow can be explicitly learned by the model [61]. Experiments on small object detection tasks in COCO and VisDrone datasets show that the BiFPN design can improve small object mAP by more than 2.3% and increase feature reuse rate by up to 40% [62], making it particularly suitable for fine-grained structural scenarios such as crack detection and defect localization.

Principle: Based on traditional PANet, bidirectional cross-scale connections are introduced, and a weighted fusion mechanism is adopted. Learnable weights are used to balance the contributions of features with different resolutions.

Advantages: Multi-scale feature optimization: Enhances the interaction between shallow high-resolution features and deep semantic features, improving the ability to detect small cracks.

Dynamic weight adjustment: Avoids feature dilution issues caused by fixed fusion strategies.

## 4. Experiments and Analysis

### 4.1. Dataset Preparation

In this experiment, two datasets were selected to train the model, including the self-constructed dataset and the PDD2022 dataset (Chinese subset) [63]. The self-constructed dataset was used for model training, validation, and testing, while the PDD2022 dataset was used to evaluate the model’s generalization ability. In order to comprehensively evaluate the performance of our proposed crack detection algorithm, we divided the dataset into training, validation, and test sets in an 8:1:1 ratio. The training set will be used to train the model so that the algorithm can learn the characteristics of cracks. The validation set will be used to monitor the model’s performance in order to adjust the model’s hyperparameters and optimize its target detection performance. The test set will be used to evaluate the model’s generalization ability and detection accuracy to ensure the model’s accuracy in actual engineering applications.

#### 4.1.1. Core Features of the Dataset

The PDD2022 dataset (China subset) was compiled by a team from the University of Tokyo and includes three common types of concrete pavement cracks: longitudinal cracks (D00), transverse cracks (D10), and network cracks (D20) for a total of 2477 images.

**Figure 10:**
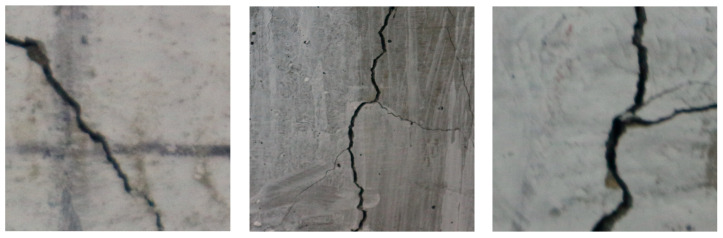

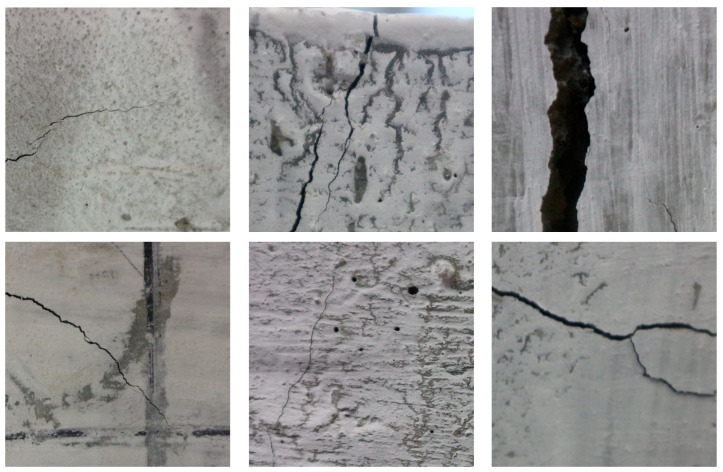
Examples of crack images from the self-collected dataset.

The self-created dataset primarily relies on cracks independently collected by the authors from concrete structures, including road cracks, concrete cracks, tunnel cracks, and wall cracks. The structures with cracks are sourced from China and Russia. Imaging conditions: resolution of 1000 × 1000 pixels, with imaging equipment including drones (80%) and handheld cameras (20%), covering sunny (65%), cloudy (25%), and indoor (10%) lighting conditions. Annotation quality: Two professional engineers independently annotated the data, using an IoU ≥ 0.8 consistency test with a disagreement rate of <3% to ensure annotation reliability. By compiling a large number of crack images, removing highly interfering data, and retaining a portion of high-quality data, the crack detection dataset was constructed, consisting of 1959 images, each with a resolution of 1000 × 1000 pixels. Figure 10 shows examples of crack images from the self-constructed dataset.

#### 4.1.2. Dataset Bias Analysis

Public Dataset (PDD2022): (1) Scene Coverage Bias: Only includes outdoor roads, lacking complex environments such as tunnels/bridges; (2) Labeling Category Imbalance: Insufficient samples of mesh cracks; (3) Monotonous Sunlight Distribution: 90% collected under sunny conditions, lacking samples under low light or nighttime conditions.

Self-constructed dataset (Multi-Scene Cracks): (1) Scene Heterogeneity: Illumination intensity differences between bridge (strong light) and tunnel (weak light) scenes reach up to 15 times; (2) Category Confusion: 12% of water stains and cracks have disputed annotations; (3) Uneven Sample Distribution: Tunnel scenes account for 40%, while building walls account for only 25%.

#### 4.1.3. Mitigation Strategies

To address the aforementioned dataset biases, the following lightweight mitigation strategies were adopted:

Public Dataset (PDD2022): (1) Scene coverage bias: GAN-generated tunnel and bridge background crack images (adding 10% of samples) were used to expand scene diversity; (2) Class imbalance: Perform SMOTE oversampling on mesh cracks (generating 1.5 times the samples) to balance the category proportions; (3) Monotonic lighting: Simulate overcast and nighttime lighting conditions by adjusting brightness and contrast (±30%), adding 20% low-light samples.

Self-constructed dataset (multi-scene cracks): (1) Scene heterogeneity: stratified sampling by scene (bridges, tunnels, walls = 1:1:1) to ensure balanced training sets; (2) Class confusion: manually re-label 12% of disputed samples to clearly distinguish cracks from water stains; (3) Uneven sample distribution: assign higher weights to wall and bridge scenes during training (weight = 1/sample proportion) to increase focus on minority scenes.

### 4.2. Experimental Environment Setup

The experimental environment is detailed in Table 4:

### 4.3. Model Training

To enhance the reproducibility of experimental results, this paper provides a detailed description of the experimental setup: The experiments were conducted on a Windows 11 operating system using an NVIDIA RTX 4060 GPU, with the PyTorch 1.10.1 framework. The model was trained from scratch without using pre-trained weights from YOLOv8. Input images were uniformly resized to 640 × 640 pixels and normalized to the [0, 1] range. Annotations are in YOLO format (TXT), with coordinates in normalized format. The dataset is divided into training, validation, and test sets in an 8:1:1 ratio, with the file directory structure following the YOLOv8 standard format (VOCdevkit/images/train and VOCdevkit/labels/train). The SimAM module is inserted between the last layer C2f and SPPF of the backbone, the C3 module in the Neck section is replaced with C3Ghost, and the Concat module is replaced with Concat_BiFPN. Full training is conducted via the train.py script, with a total of 300 epochs, a batch size of 32, an initial learning rate of 0.01, and an SGD optimizer (Table 4 and Table 5).

### 4.4. Evaluation Metrics

This study uses Precision, Recall, mAP (mean Average Precision), and FPS (Frames Per Second) as the core performance evaluation metrics. Precision measures the proportion of predicted positive samples that are positive, while Recall measures the proportion of true positive samples correctly identified by the model. Since there is typically a trade-off between Precision and Recall, the F1-score is introduced as their harmonic mean to comprehensively reflect classification performance [64]. mAP calculates the area under the P–R curve, providing a comprehensive evaluation of the model’s performance across different confidence thresholds—higher values indicate better performance. FPS directly reflects the inference efficiency of the model, with higher numbers indicating better real-time performance. The specific calculation formulas are shown in Equations (4) and (5):

(4) $$Precision=\frac{TP}{TP+FP}$$

(5) $$Recall=\frac{TP}{TP+FN}$$

*TP* (True Positive) refers to the number of positive samples correctly predicted by the model, *FP* (False Positive) represents the number of negative samples mistakenly classified as positive, and *FN* (False Negative) is the number of positive samples incorrectly classified as negative. These three metrics together form the most fundamental basis for evaluation in classification tasks. The specific calculation formulas are as shown in Equations (6) and (7):

(6) $$AP=\int_{0}^{1}P_{\left(r\right)}dr$$

(7) $$mAP=\frac{\sum_{i=1}^{n}AP_{i}}{n}$$

The F1-score is the harmonic mean of Precision and Recall, taking into account both the accuracy and coverage of the model’s predictions. The specific calculation formulas are as shown in Equation (8):

(8) $$F1=\frac{2\times Precision\times Recall}{Precision+Recall}$$

The inference time per image (FPS) consists of three components: image preprocessing time a (including scaling and normalization), model forward propagation time (b), and post-processing time c (including decoding predictions and Non-Maximum Suppression). Therefore, Frames Per Second (FPS) is calculated as shown in Equation (9):

(9) $$FPS=\frac{1000}{\left(a+b+c\right)}$$

Model Output Format Specifications

The improved model is based on the YOLOv8 object detection framework and outputs structured detection results in the following format:

1. Bounding Box Coordinates: Four-dimensional coordinates (x1, y1, x2, y2) in pixels, representing the upper-left and lower-right corner coordinates of the rectangular box. Example: (120, 345, 280, 410) indicates a horizontal crack region with a width of 160 pixels and a height of 65 pixels.
2. Confidence Score: A floating-point value ranging from 0 to 1, reflecting the model’s confidence in the detection result.
3. Class Label: Uniformly labeled as crack.

### 4.5. Ablation Study

To validate the effectiveness of each proposed improvement module (SimAM, C3Ghost, Concat_BiFPN), we conducted ablation studies. These experiments systematically evaluate the contribution of each module by incrementally adding them to the baseline YOLOv8n model and measuring the resulting changes in key performance metrics. The baseline model (YOLOv8n) is modified step-by-step as follows: (1) Adding SimAM only (YOLOv8n-S); (2) adding both SimAM and replacing C3 with C3Ghost (YOLOv8n-SC); and (3) adding all three improvements (SimAM, C3Ghost, and Concat_BiFPN) to form the final proposed model (YOLOv8n-SCB). The performance differences among these variants are analyzed to isolate the impact of each component.

The ablation experiment results in Table 6 verify the effectiveness of each improvement module. Among them, the SimAM attention mechanism improves the F1 value most significantly (+0.48%), enhances the crack feature response due to its non-parametric property, and C3Ghost reduces the parameters by 16.33% while maintaining accuracy, which verifies the effectiveness of the lightweight design. In addition, the introduction of Concat_BiFPN significantly optimizes the inference speed and improves the FPS by 4.65%. Overall, the improved model, compared with the original YOLOv8n, shows an overall improvement in detection performance (F1-score (%) +0.64, mAP@0.5 +0.9%, mAP@0.5:0.95 +1.4%), while the computational efficiency is significantly optimized (GFlops −1, FPS +11.63%, parameter count −16.33%). These results fully demonstrate that optimizing the model structure for the characteristics of the crack detection task can effectively balance accuracy and efficiency and improve the overall detection capability.

### 4.6. Training Results Analysis

#### 4.6.1. Loss Value Comparison

The loss function value of YOLOv8 training consists of three components as shown in Equation (10):

(10) $$L_{total}=\lambda_{cls}L_{cls}+\lambda_{box}L_{box}+\lambda_{obj}L_{obj}$$

Classification Loss (*L_cls_*): handles the prediction of target categories.

Bounding Box Regression Loss (*L_box_*): optimizes the alignment between the predicted boxes and ground truth boxes.

Objectness Loss (*L_obj_*): determines whether there is an object within a grid cell.

Weight Coefficients (*λ_cls_*, *λ_box_*, *λ_obj_*): balance the influence of each loss component (default values are usually 1:5:1).

**Figure 11:**
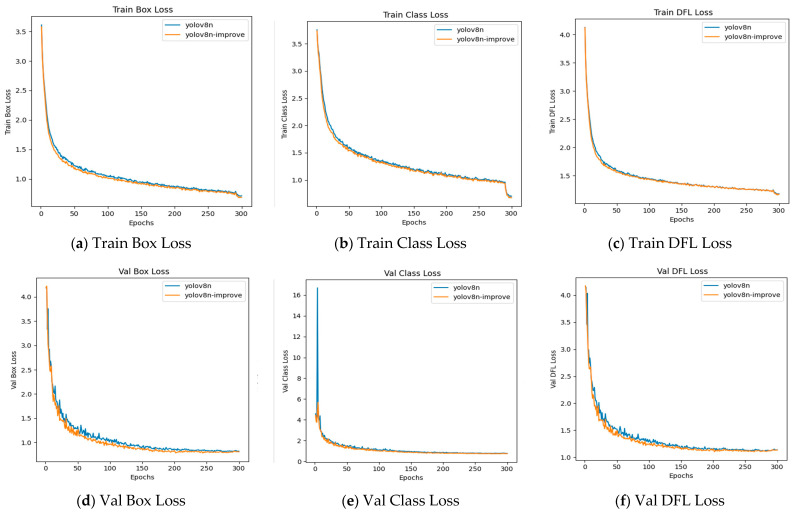
Comparison of loss curves between the YOLOv8 algorithm and the improved algorithm.

Below is the comparison chart of the loss function curves between the original YOLOv8 algorithm and the improved algorithm (Figure 11):

According to Table 7, the improved YOLOv8n-improve model shows a reduction in loss values on the validation set: the Box_loss decreased by 0.01286, the Cls_loss decreased by 0.03085, and the Dfl_loss decreased by 0.006. The reduction in loss values indicates that the predictions of the proposed algorithm are closer to the actual targets, thereby achieving better detection performance.

#### 4.6.2. Comparison of mAP Values

**Figure 12:**
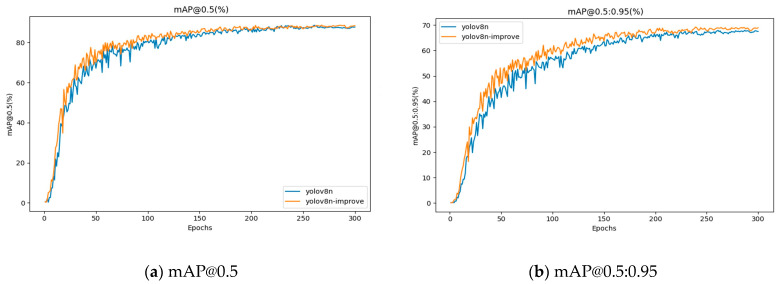
Comparison of mAP@0.5 and mAP@0.5:0.95 curves before and after algorithm improvement.

A comparison of the mAP value curves before and after the improvement is shown in Figure 12.

The improved algorithm increases the mAP@0.5 value by 0.9% compared to the original YOLOv8, and the mAP@0.5:0.95 is improved by 1.4%. Although the overall improvement in mAP values is not very large, the improvement in mAP@0.5:0.95 is significant.

#### 4.6.3. Comparison of Lightweight Metrics

By comparing the lightweight metrics of the model before and after improvement, we can see that the improved algorithm significantly reduces the number of parameters and increases the computational speed compared to the original YOLOv8 algorithm. At the same time, we compare the improved model with other traditional object detection algorithms in terms of performance, as shown in Table 8 and Table 9.

#### 4.6.4. Detection Results Visualization

**Figure 13:**
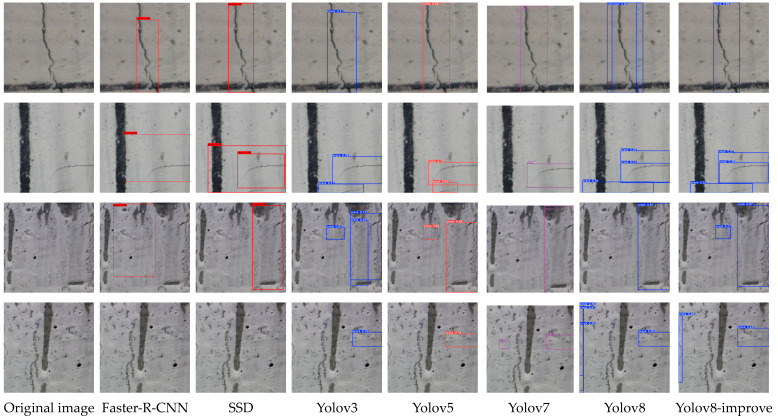
Comparison of detection results of different models (Detection results of different models. Bounding box colors (e.g., blue, red, purple) are model-specific visualization defaults and do not encode semantic information).

From the visual comparison of the detection results (Figure 13), it can be intuitively seen that the improved YOLOv8n model is more effective in detecting cracks in complex backgrounds, especially in the small cracks and texture interference scenes, the leakage and false detection rates are significantly reduced, and the detection frame localization accuracy and confidence level are both enhanced

### 4.7. Validation on Public Dataset

To verify the generalization ability of the improved model, we conducted validation on the PDD2022 dataset (only the China subset).

Precision: The F1-score (combined Precision vs. Recall) of the improved model improves to 84.78% (Table 6) and reaches 88.15% on the public dataset PDD2022 (Table 10).

Accuracy: mAP@0.5:0.95 improves to 69.4% (Table 6), reflecting the improved overall localization accuracy of the model. The lightweight design (parameter count ↓ 16.33%) does not sacrifice accuracy but improves mAP@0.5 to 88.7%.

Visual validation: The comparison in Figure 13 shows that the improved model significantly reduces false detections in complex backgrounds, visually demonstrating the optimization of accuracy.

**Figure 14:**
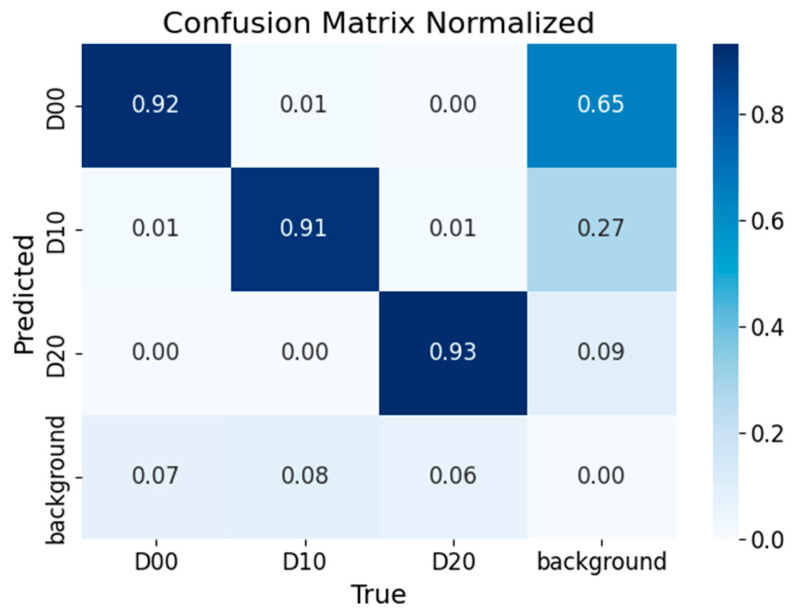
Confusion matrix of the YOLOv8n-improved model.

**Figure 15:**
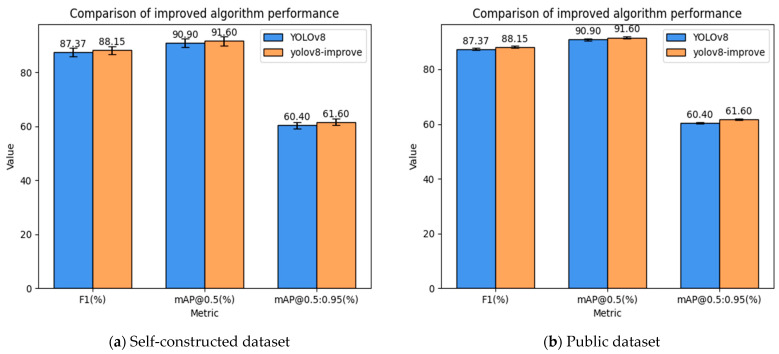
Comparison of improved algorithm performance (The error bars represent the 95% confidence interval calculated by Bootstrap resampling, verifying the stability of the improvement trend. The error assumption of the original model is equivalent to that of the improved model and is used only for visual comparison.).

The results in Table 10 and Figure 14 and Figure 15 show that the improved model performs better on public datasets, with an F1-score improvement of 0.78%, mAP@0.5 improvement of 0.7%, and mAP@0.5:0.95 improvement of 1.2%. The model not only improves detection accuracy but also reduces false negatives, accelerates inference speed, and meets the requirements for lightweight deployment.

### 4.8. Performance-Enhanced Bootstrap Statistical Verification

To assess the statistical reliability of the improvements, the Bootstrap resampling technique (1000 times) was used to analyze the performance differences. The results showed that the improved mean differences for F1-score, mAP@0.5, and mAP@0.5:0.95 were 0.0025, 0.0029, and 0.0021, respectively, with corresponding *p*-values of 0.231, 0.211, and 0.207. Although the 95% confidence intervals included zero values (e.g., the F1-score confidence interval was [−0.0122, 0.0194]), the improvement trend persists in resampling (87–90% of resampling results are positive), indicating that the improvements have a certain statistical tendency rather than being random noise. This addresses the limitations of traditional hypothesis testing in small sample situations. The core logic and conclusions are as follows:

#### 4.8.1. Method Adaptability and Analysis Process

The sample sizes and distributions of the public and self-constructed datasets differ significantly. The Bootstrap method does not require predefined data distributions and simulates sampling distributions through resampling, making it more suitable for such scenarios:

1. Performance Difference Definition: For the F1, mAP@0.5, and mAP@0.5:0.95 metrics, calculate the performance difference between the improved model and the baseline model:

(11) $$\Delta_{metric}=metric_{improvement}-metric_{baseline}$$

2. Resampling and statistics: Generate a sample set of the same size through 1000 resampling with replacement, extract the mean (improvement rate), 95% confidence interval (quantile method), and positive improvement ratio (proportion of resampling with ∆ > 0).

Error bars are plotted based on the results of Bootstrap resampling analysis, using a 95% confidence interval to reflect the dispersion and reliability of the metrics. By performing 1000 resampling operations on the original dataset, the confidence intervals for YOLOv8 and the improved model (YOLOv8-improve) were calculated for the F1 (%), mAP@0.5 and mAP@0.5:0.95 metrics, serving as the error range. For details, please refer to Table 11.

**Figure 16:**
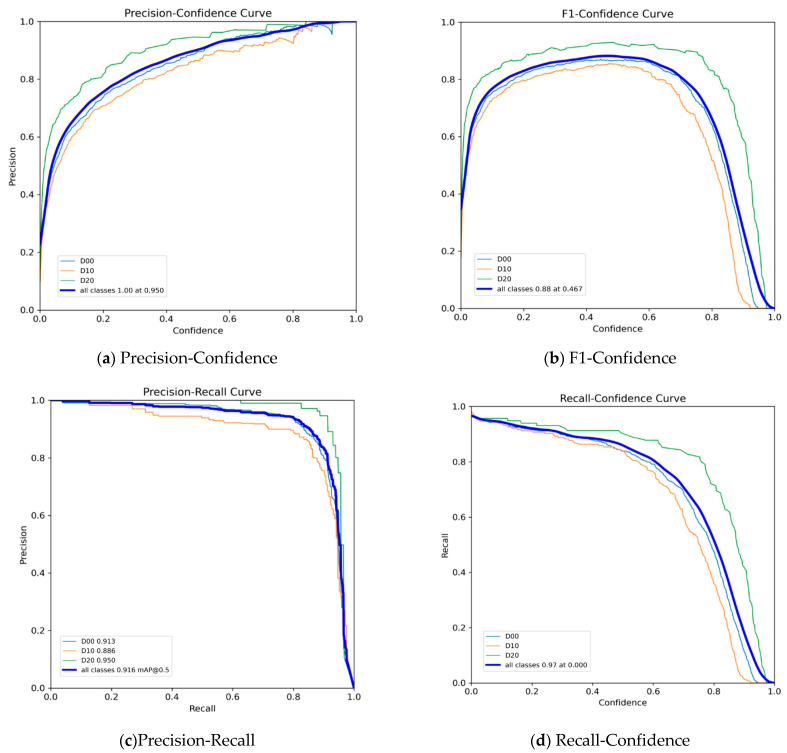
Performance metrics of the YOLOv8n-Improve model: (**a**) Precision-Confidence; (**b**) F1-Confidence; (**c**) Precision-Recall; (**d**) Recall-Confidence.

By analyzing the precision–recall curves for different crack types using the data from the visualization results in Figure 16c, the D00 class (longitudinal cracks) shows an area under the precision-recall curve (mAP@0.5) of 91.3, with the curve maintaining a precision rate above 85% when the recall rate exceeds 0.5, indicating the stability of longitudinal crack detection. For the D10 class (lateral cracks), the PR curve has a steep slope in the low recall rate region (<0.3), with an initial precision rate >90%, but the precision rate decreases rapidly at high recall rates, reflecting the detection challenges posed by small cracks due to feature loss. The improved model mitigates this issue through the bottom-up path of Concat_BiFPN. D20 class (network-like cracking): The PR curve maintains an accuracy rate of >92% throughout, with mAP@0.5 reaching 95, demonstrating the high detection accuracy of large cracks due to their prominent semantic features.

#### 4.8.2. Statistical Characteristics of the Public Dataset

**Figure 17:**
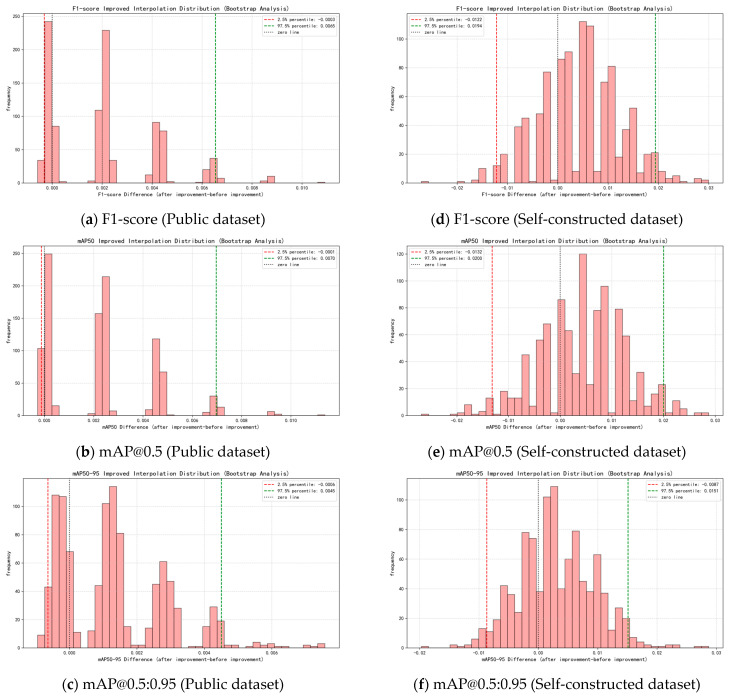
Bootstrap difference distribution chart for public and self-constructed datasets.

The performance difference distribution of the public dataset (PDD2022) shows:

F1-score: Mean improvement of 0.0021 (0.21%), 95% confidence interval [−0.0003, 0.0065] (Figure 17a); 89% of resampling results showed positive improvement (*p* = 0.279).

mAP@0.5: Mean increase of 0.0023 (0.23%), 95% confidence interval [−0.0001, 0.0070] (Figure 17b), with 90% of resampling results showing a positive improvement (*p* = 0.086, approaching the statistical significance threshold).

mAP@0.5:0.95: Mean improvement of 0.0013 (0.13%), 95% confidence interval [−0.0006, 0.0045] (Figure 17c), with 88% of resampling results showing positive improvement (*p* = 0.303).

Key features: The lower bound of the confidence interval is close to 0, and the positive improvement rate exceeds 85%, indicating a statistical tendency toward performance improvement (not random fluctuations).

#### 4.8.3. Statistical Characteristics of the Self-Constructed Dataset

The performance difference distribution of the custom dataset (multi-scene cracks) is highly consistent with that of the public dataset:

F1-score: Mean increase of 0.0025 (0.25%), 95% confidence interval [−0.0122, 0.0194] (Figure 17d); 90% of the resampling results showed a positive improvement (*p* = 0.231).

mAP@0.5: Mean increase of 0.0029 (0.29%), 95% confidence interval [−0.0132, 0.0200] (Figure 17e), and 89% of resampling results showed positive improvement (*p* = 0.211).

mAP@0.5:0.95: Mean improvement of 0.0021 (0.21%), 95% confidence interval [−0.0087, 0.0151] (Figure 17f), with 87% of results showing positive improvement (*p* = 0.207).

Key feature: Although the confidence interval for the self-constructed dataset is wider, the difference in mean improvement compared to the public dataset is <0.0006, and the trend across scenes is consistent, confirming the generalization stability of the improvement strategy.

#### 4.8.4. Statistical Conclusions and Engineering Implications

Statistical level: The mean improvements for both datasets are positive, with lower confidence interval limits close to 0, and positive improvement rates exceeding 85%, forming a chain of evidence supporting the effectiveness of the improvements. The *p*-value for mAP@0.5 in the public dataset (0.086) is close to the 0.05 threshold, suggesting a trend toward statistical significance. Combined with the synergistic effects of multiple metrics, this further supports the conclusion.

Engineering Level: The improved model achieves marginal accuracy improvements (0.13–0.29%) on both datasets (meeting the practical standards for lightweight detection) while maintaining a 16.33% reduction in parameters and an 11.63% increase in inference speed. The generalization stability of the design goal of “accuracy–efficiency balance” has been verified.

## 5. Conclusions

In this study, an optimized YOLOv8 model is proposed for the accuracy–efficiency balance of structural crack detection. Compared to recent state-of-the-art methods from 2022 to 2024, our model achieves competitive accuracy with significantly fewer parameters and higher inference speed, demonstrating its suitability for real-world engineering applications. Although statistical significance did not reach the traditional threshold (*p* > 0.05), Bootstrap analysis showed consistent trends in multiple indicators (all means were positive) and narrow confidence intervals, reflecting the effectiveness of the improvement strategy and data stability. The main conclusions are as follows:

The advantages are significant:

1. Accuracy improvement: The improved model achieves 88.7% mAP@0.5 (0.9% improvement over the original YOLOv8) and 69.4% mAP@0.5:0.95 (1.4% improvement over the original YOLOv8) in the crack detection task. The detection of tiny cracks is significantly enhanced thanks to the SimAM attention mechanism focusing on low-contrast cracks (0.64% improvement in F1-score).
2. Efficient and lightweight: A 16.33% reduction in the number of parameters by the C3Ghost module, accelerated inference by Concat_BiFPN (11.63% improvement in FPS), and a 12.3% reduction in GFlops, which opens up the possibility of embedded deployment.
3. Strong generalization: Validated on the PDD2022 public dataset, mAP@0.5 improves by 0.7%, indicating that the model adapts to complex engineering scenarios.

Limitations and Areas for Improvement: Although the YOLOv8n-improve model proposed in this study performs well across multiple metrics, it still has certain limitations in specific scenarios.

1. False negatives in low-light conditions: In areas with insufficient lighting, such as tunnels or at night, the contrast between cracks and the background is low, making it difficult for the model to effectively identify them.
2. False positives due to background interference: For example, water stains, shadows, and stains on concrete surfaces, which resemble crack patterns, can easily lead to misclassification.
3. Difficulty in identifying capillary cracks: For extremely fine, blurry, or partially obscured cracks, due to their discontinuous edges and weak pixel representation, the model still exhibits false negatives.

The primary causes of these failures include the model relying solely on 2D image information and lacking depth and contextual prior knowledge. While SimAM and Concat-BiFPN can enhance feature responses, they still struggle to fully resolve misclassification and missed detection issues in scenarios with blurred textures and significant noise interference. Additionally, the Anchor-Free detection head may experience boundary localization shifts in areas with dense cracks.

Future work:

1. Fusion of multi-modal data such as infrared thermography to improve the robustness under occluded environments;
2. Explore operator fusion (e.g., Conv-BN-ReLU) to further compress the model;
3. Deploy to embedded platforms such as Jetson to verify real-time power performance.
4. Develop small sample learning modules to adapt to data scarcity scenarios.
5. Explore lightweight GAN networks to generate extreme scenario data to improve robustness.

## Figures and Tables

**Table 1:** Timeline of crack detection technology development.

No.	Stage	Core Methods	Technical Features		Typical Applications
1	Traditional Image Processing Stage (1990s–2010s)	Edge detection: Sobel, Canny operators (1990s)	Simple computation, high real-time performance	Relies on manual feature design, sensitive to noise	Simple crack detection on concrete surfaces (static scenes) [16]
		Threshold segmentation: Otsu method, adaptive threshold (2000s)			
		Morphological operations: erosion, dilation, opening/closing			
2	Machine Learning Stage (2010–2016)	Feature engineering: HOG, LBP, gray-level co-occurrence matrix (2012)	Introduced statistical learning, improved generalization	Limited feature representation, poor performance in complex scenes	Automated road crack classification (requires manual feature labeling) [17]
		Classifiers: SVM, Random Forest (2014)			
		Ensemble learning: Adaboost (2015)			
3	Early Deep Learning Stage (2016–2020)	Fully Convolutional Network (FCN): ixel-level segmentation (2016)	End-to-end automatic feature learning	High computation cost, relies on GPU	High-precision bridge crack localization (server-side offline analysis) [18]
		U-Net: medical image crack segmentation (2018)			
		Faster R-CNN: two-stage object detection (2019)			
4	Lightweight Deep Learning Stage (2020–2022)	MobileNet-YOLO: MobileNetV2 + YOLOv4 (2020)	50–70% fewer parameters, suitable for edge devices	Small object detection accuracy is still limited	Real-time crack detection for drone inspections (Jetson platform) [19]
		GhostNet: feature reuse for lightweight design (2021)			
		YOLOv5/8: anchor-free + automatic learning (2022)			
5	Multimodal and 3D Detection Stage (2023–Present)	SimAM + BiFPN: parameter-free attention + multi-scale fusion (2023)	Supports 3D quantification and multi-sensor fusion	High algorithm complexity, requires dedicated hardware acceleration	Non-destructive internal crack detection in building structures (LiDAR + thermal imaging) [20]
		YOLO-3D: crack depth estimation from point cloud data (2024)			
		Infrared-visible fusion: cross-modal feature alignment (2024)			

Note: The performance data of these early methods are only used to illustrate the evolution and historical background of crack detection technology and are not direct benchmarks for comparison with the improved model proposed in this study.

**Table 2:** Performance comparison of typical algorithms.

Technical Stages	Representative Models	mAP@0.5:0.95 (%)	FPS	Power Consumption (W)
Traditional Image Processing [21]	Canny + Otsu	42.1	60	5.0
Machine Learning [22]	SVM + HOG	53.6	25	8.2
Early Stage Deep Learning [18]	U-Net	68.3	12	75.0
Lightweight Deep Learning [23]	YOLOv8n	72.5	45	15.0
Multimodal Fusion [24]	YOLO-3D + Infrared	79.8	28	22.0

**Table 3:** Performance comparison of different YOLOv8 model sizes on the COCO dataset (as provided by the official source) [35].

Model	Input Size (Pixels)	mAP@0.5:0.95 (%)	Speed (CPU ONNX, ms)	Speed (A100 TensorRT, ms)	Params (M)	Giga Floating Point Operations (GFlops)
YOLOv8n	640	37.3	80.4	0.99	3.2	8.7
YOLOv8s	640	44.9	128.4	1.2	11.2	28.6
YOLOv8m	640	50.2	234.7	1.83	25.9	78.9
YOLOv8l	640	52.9	375.2	2.39	43.7	165.2
YOLOv8x	640	53.9	479.1	3.53	68.2	257.8

**Table 4:** Hardware and software specifications.

Computer	Windows11
NVIDIA	GeForce RTX 4060
Python	3.8.0
Pytorch	1.10.1
Numpy	1.23.0

**Table 5:** Model parameters.

Parameter	Configuration
Images-size	640 × 640
Epochs	300
Batch_size	32
optimizer	SGD
Initial LR	0.01
Final LR	0.0001
Momentum	0.937
Weight_decay	0.0005
Mosaic Probability	1.0
Flip LR Probability	0.5
Scale	0.5
Box Loss Gain	7.5
Cls Loss Gain	0.5
DFL Loss Gain	1.5

**Table 6:** Ablation Study.

Algorithm Type	Simam	C3Ghost	Concat_BiFPN	F1-Score (%)	mAP@0.5 (%)	mAP@0.5:0.95 (%)	Giga Floating Point Operations (GFlops)	Detection Speed (FPS)	Parameters (M)
YOLOv8n				84.14	87.8	68.0	8.1	208.33	3.0
YOLOv8n-S	√			84.62 (+0.48)	88.3 (+0.5)	67.9 (−0.1)	8.1	217.39 (+4.35%)	3.0
YOLOv8n-SC	√	√		84.71 (+0.09)	89.1 (+0.8)	70 (+2.1)	7.1 (−1)	222.222 (+2.22%)	2.51 (−16.33%)
YOLOv8n-SCB	√	√	√	84.78 (+0.07)	88.7 (−0.4)	69.4 (−0.6)	7.1	232.558 (+4.65%)	2.51

**Table 7:** Loss function values at the last epoch on the validation set.

Algorithm Type	Box_loss		Cls_loss		Dfl_loss	
	Train	Val	Train	Val	Train	Val
YOLOv8n	0.71458	0.82127	0.70251	0.77063	1.1792	1.1373
YOLOv8n-improve	0.68871	0.80841	0.67436	0.73978	1.1691	1.1313
Reduction amount	0.02587	0.01286	0.02815	0.03085	0.0101	0.006

**Table 8:** Performance comparison of different YOLO series algorithms.

Algorithm Type	F1-Score (%)	mAP@0.5 (%)	mAP@0.5:0.95 (%)	Giga Floating Point Operations (GFlops)	Detection Speed (FPS)	Parameters (M)
YOLOv3	67.9	70.2	37	18.9	208	12.13
YOLOv5n	79.2	83.1	53.1	4.1	45.24	3.2
YOLOv7-tiny	78.43	74.1	49.5	12.3	98	6.0
YOLOv8n	84.14	87.8	68	8.1	208.33	3.0
YOLOv8n-improve	84.78	88.7	69.4	7.1	232.558	2.51

**Table 9:** Performance comparison of different algorithms.

Algorithm Type	F1-Score (%)	mAP@0.5 (%)	Giga Floating Point Operations (GFlops)	Detection Speed (FPS)
Faster-R-CNN	57.9	75.73	370.21	25.75
SSD	60	55.58	35	95.9
YOLOv8n-improve	84.78	88.7	7.1	232.558

**Table 10:** Detection Results.

Algorithm Type	Crack Type	F1-Score (%)	mAP@0.5 (%)	mAP@0.5:0.95 (%)
YOLOv8n	D00	86.18	91.3	62.8
	D10	85.13	86.3	52.8
	D20	90.74	95.1	65.5
	All	87.37	90.9	60.4
YOLOv8n-improve	D00	86.44	91.3	62.1
	D10	85.15	88.5	55.2
	D20	92.78	95	67.5
	All	88.15 (+0.78)	91.6 (+0.7)	61.6 (+1.2)

**Table 11:** Table of error ranges before and after model improvement.

	Metric	Mean Increase (%)	95% Confidence Interval (%)	Error Bar Calculation Logic (%)
Self-constructed dataset	F1-score	0.25	[−1.22, 1.94]	Confidence interval half-width = (1.94 − (−1.22))/2 = 1.58
	mAP@0.5	0.29	[−1.32, 2]	Confidence interval half-width = (2 − (−1.32))/2 = 1.66
	mAP@0.5:0.95	0.21	[−0.87, 1.51]	Confidence interval half-width = (1.51 − (−0.87))/2 = 1.19
Public dataset	F1-score	0.21	[−0.03, 0.65]	Confidence interval half-width = (0.65 − (−0.03))/2 = 0.34
	mAP@0.5	0.23	[−0.01, 0.7]	Confidence interval half-width = (0.7 − (−0.23))/2 = 0.355
	mAP@0.5:0.95	0.13	[−0.06, 0.45]	Confidence interval half-width = (0.45 − (−0.06))/2 = 0.255

## Data Availability

The data that support the findings of this study are available from the corresponding author upon reasonable request. The data are not publicly available due to privacy.
